# Chemical composition, antinociceptive, anti-inflammatory and redox properties *in vitro* of the essential oil from *Remirea maritima* Aubl. (Cyperaceae)

**DOI:** 10.1186/1472-6882-14-514

**Published:** 2014-12-23

**Authors:** Alessandra Silva Rabelo, Mairim Russo Serafini, Thallita Kelly Rabelo, Marcelia Garcez Dória de Melo, Douglas da Silva Prado, Daniel Pens Gelain, José Claudio Fonseca Moreira, Marília dos Santos Bezerra, Thanany Brasil da Silva, Emmanoel Vilaça Costa, Paulo Cesar de Lima Nogueira, Valéria Regina de Souza Moraes, Ana Paula do Nascimento Prata, Lucindo José Quintans, Adriano Antunes Souza Araújo

**Affiliations:** Laboratório de Ensaios Farmacêuticos e Toxicidade, Universidade Federal de Sergipe (LeFT/UFS), 49100-000 São Cristóvão, Sergipe Brazil; Centro de Estudos em Estresse Oxidativo, Departamento de Bioquímica, Instituto de Ciências Básicas da Saúde, Universidade Federal do Rio Grande do Sul, 13083-970 Porto Alegre, Rio Grande do Sul Brazil; Laboratório de Pesquisa em Química Orgânica de Sergipe (LABORGANICS), Departamento de Química, Universidade Federal de Sergipe, 49100-000 São Cristóvão, Sergipe Brazil; Laboratório de Sistemática Vegetal, Departamento de Biologia, Universidade Federal de Sergipe, 49100-000 São Cristóvão, Sergipe Brazil

**Keywords:** *Remirea maritima*, Essential oil composition, Redox properties

## Abstract

**Background:**

The present study was carried out to evaluate antioxidant, antinociceptive and anti-inflammatory activities of essential oil from *R. maritima* (RMO) in experimental protocols.

**Methods:**

The essential oil from the roots and rhizomes of RMO were obtained by hydrodistillation using a Clevenger apparatus, and analyzed by gas chromatography/mass spectrometry (GC/MS). Here, we evaluated free radical scavenging activities and antioxidant potential of RMO using *in vitro* assays for scavenging activity against hydroxyl radicals, hydrogen peroxide, superoxide radicals, and nitric oxide. The total reactive antioxidant potential (TRAP) and total antioxidant reactivity (TAR) indexes and *in vitro* lipoperoxidation were also evaluated. The ability of RMO to prevent lipid peroxidation was measured by quantifying thiobarbituric acid-reactive substances (TBARS). NO radical generated at physiological pH was found to be inhibited by RMO, that showed scavenging effect upon SNP-induced NO production at all concentrations. Antinociceptive and anti-inflammatory properties were evaluated by acetic acid writhing reflex, Formalin-induced nociception and Carrageenan-induced edema test.

**Results:**

The majors compounds identified was remirol (43.2%), cyperene (13.8%), *iso*-evodionol (5.8%), cyperotundone (5.7%), caryophyllene oxide (4.9%), and rotundene (4.6%). At the TRAP assay, RMO concentration of 1 mg.mL^−1^ showed anti-oxidant effects and at concentration of 1 and 10 ng.mL^−1^ RMO showed pro-oxidant effect. RMO at 1 mg.mL^−1^ also showed significant anti-oxidant capacity in TAR measurement. Concentrations of RMO from 1 ng.mL^−1^ to 100 μg.mL^−1^ enhanced the AAPH-induced lipoperoxidation. RMO reduced deoxyribose oxidative damage, induced by the Fenton reaction induction system, at concentrations from 1 ng.mL^−1^ to 100 μg.mL^−1^. We observed that RMO caused a significant increase in rate of adrenaline auto-oxidation. On the other hand RMO did not present any scavenging effect in H_2_O_2_ formation *in vitro*. The results of this study revealed that RMO has both peripheral and central analgesic properties. The RMO, all doses, orally (p.o.) administered significantly inhibited (p < 0.05, p < 0.01 and p < 0.001) the acetic acid-induced writhings and two phases of formalin-induced nociception in mice.

**Conclusion:**

The RMO demonstrated antioxidant and analgesic profile which may be related to the composition of the oil.

**Electronic supplementary material:**

The online version of this article (doi:10.1186/1472-6882-14-514) contains supplementary material, which is available to authorized users.

## Background

The use of plants as a source of products for the treatment of diseases that affect mankind dates back to ancient ages. However, there are few plant species present in the literature with specifications for assessing the quality of the originating raw materials as well as validation methods that can be used for quantification of chemical markers which is essential for guaranteeing the efficacy and safety of the derived products [1]. In the last decades, the essential oils and various extracts of plants have been of great interest as they have been the sources of natural products [2].

Essential oils are mixture of volatile compounds (mainly mono- and sesquiterpenoids, benzenoids, amino acid- derivatives, phenypropanoids, etc.) with biological function on humans, animals, and other plants [3]. Nowadays, essential oils are not only popular as ingredients of perfumes, cosmetics, household cleaning products, foods and beverages, but because of its medicinal applications they have become useful in the treatment of different diseases due to their anticancer, anticonvulsant, anti-inflammatory, antimicrobial, analgesic, and antioxidant properties [4].

In recent years, there is an increasing interest in finding antioxidant phytochemicals, because they can inhibit the propagation of free radical reactions, protect the human body from diseases [5] and retard lipid oxidative rancidity in foods [6]. Free radicals and other reactive oxygen species (ROS), such as superoxide anion, hydroxyl radical, and hydrogen peroxide are an entire class of highly reactive molecules derived from the normal metabolism of oxygen or from exogenous factors and agents. Oxidative damage to crucial cellular molecules induced by ROS has been implicated as a possible factor in the etiology of several human diseases, including cancer, cardiovascular disease, and aging [7]. Another approach to natural product with antioxidant property is study in pain models [8].

In this context, plants have attracted the interest of many researchers and the pharmaceutical industry itself for being able to produce, transform, and accumulate numerous other substances, not necessarily directly related to the maintenance of cellular metabolism, called secondary metabolites [9]. Among the plants with promising biological activities based on the traditional use by the population, *Remirea maritima* Aubl. stands out, though studies involving this species are scarce in current literature. *R. maritima* Aubl. [Syn. *Mariscus pedunculatus* (R. Br.) T. Koyama] is a tropical Cyperaceae species popularly known as “capim-da-praia”, which has been used traditionally to treat diarrhea, kidney disease, high fever, pain, and inflammations [10].

Previous reports on *R. maritima* include the isolation of flavones glycosides, cyperaquinones and phenolic ketones from the roots and rhizomes of the Cyperaceae [10–13], besides their antimicrobial activity of the dihydrocyperaquinone [12] and anti-inflammatory activity of the essential oil and the remirol [11]. Recently, we have shown that the aqueous extract of *R. maritima* features consistent analgesic and anti-inflammatory effects [13]. However, little is known through scientific literature about the pharmacological effects of the oil of this plant. In this sense, the study aimed to evaluate antioxidant, antinociceptive and anti-inflammatory activities of essential oil from *R. maritima* (RMO) in experimental protocols.

## Methods

### Plant material

*R.maritima* was collected in Pirambu city, Sergipe State, Brazil (10°55´S, 35°6´W), and was identified by Dr. Ana Paula do Nascimento Prata (a plant taxonomist of the Department of Biology of the Federal University of Sergipe, São Cristovão, SE, Brazil). A voucher specimen has been deposited in the Herbarium of the Federal University of Sergipe (ASE/UFS) under N^o^ 19013.The plant was collected in October 2010 and extracted few hours after collection.

### Hydrodistillation of the rhizomes and roots

The essential oil from fresh roots and rhizomes of *R. maritima* (420 g) was obtained by hydrodistillation for 8 h using a Clevenger-type apparatus according to Siani et al. [11]. The RMO was dried over anhydrous sodium sulphate and the percentage content was calculated on the basis of the weight of plant material (317.5 mg). The essential oil was stored in a freezer until GC/MS analysis.

### GC/MS analysis

GC/MS analyses were performed on a Shimadzu QP5050A GC/MS system equipped with an AOC-20i auto-injector. A J&W Scientific DB-5MS (coated with 5%-phenyl-95%-dimethylpolysiloxane) fused capillary column (30 m × 0.25 mm × 0.25 μm film thickness) was used as the stationary phase. Helium was the carrier gas at 1.2 mL.min^−1^ flow rate. Column temperatures were programmed from 40°C for 4 min, raised to 220°C at 4°C.min^−1^, and then heated to 240°C at 20°C. min^−1^. The injector and detector temperatures were 250°C and 280°C, respectively. Samples (1.0 μL in ethyl acetate) were injected with a 1:20 split ratio. MS was taken at 70 eV with a scan interval of 0.5 s and fragments from 40–350 Da. The retention indices [14] were obtained by co-injecting the oil sample with a C_9_-C_18_ linear hydrocarbon mixture (retention index from 1800–2100 range was obtained by extrapolation). The percentage composition of each component was determined by dividing the area of the componentby the total area of all components isolated under these conditions.

The volatile components were analyzed by GC/MS, and identification was made on the basis of standard compounds co-injection and comparison of retention indices and mass spectra with those in the literature [15] as well as by computerized matching of the acquired mass spectra with those stored in the NIST and Wiley mass spectral library of the GC/MS data system and other published mass spectra.

### Isolation and identification of major compounds of the essential oil

Part of the crude essential oil (120 mg) from *R. maritima* was purified by preparative thin-layer chromatography (TLC) with hexane-EtOAc (95:5, v/v) as the mobile phase. The TLC was eluted three times to yield the fraction 1 (*iso*-evodionol (7.4 mg), fraction 2 (remirol, 15.0 mg) and fraction 3 (cyperotundone, 2.0 mg). Fractions 1–3 were analyzed by GC/MS and also by ^1^H and ^13^C NMR spectroscopy on a BRUKER DRX 400 device using CDCl_3_ as a solvent and TMS as an internal standard.

### Total reactive antioxidant potential (TRAP) and total antioxidant reactivity (TAR)

The total reactive antioxidant potential (TRAP) is employed to estimate the antioxidant capacity of samples in vitro. This method is based on the quenching of luminol-enhanced chemiluminescence (CL) derived from the thermolysis of 2,2´-azobis (2-amidinopropane) dihydrochloride (AAPH) as the free radical source [16].

The background CL was measured by adding 4 mL of AAPH (10 mM) dissolved in glycine buffer (0.1 M, pH 8.6) to a glass scintillation vial. Then, 10 μL of luminol (4 mM) was added to each vial, and the CL was measured until constant light intensity. After this stabilization time, 10 μL of Trolox solution or 10 μL of sample was added, and the CL was measured in a liquid scintillator counter working in the out of coincidence mode. The AUC was calculated using Graph Pad Prism software.

### Thiobarbituric acid reactive species (TBARS)

TBARS (thiobarbituric acid reactive species) assay was employed to quantify lipid peroxidation [17] and an adapted TBARS method was used to measure the antioxidant capacity of samples using egg yolk homogenate as lipid rich substrate [18]. Briefly, egg yolk was homogenized (1% w/v) in 20 mM phosphate buffer (pH 7.4); 1 mL of homogenate was sonicated and then homogenized with 0.1 mL of sample at different concentrations. Lipid peroxidation was induced by addition of 0.1 mL of AAPH solution (0.12 M). Control was incubation medium without AAPH. Reactions were carried out for 30 min at 37°C. Samples (0.5 mL) were centrifuged with 0.5 mL of trichloroacetic acid (15%) at 1200 g for 10 min. An aliquot of 0.5 mL from supernatant was mixed with 0.5 mL TBA (0.67%) and heated at 95°C for 30 min. After cooling, samples absorbance was measured using a spectrophotometer at 532 nm. The results were expressed as percentage of TBARS formed by AAPH alone (induced control).

### Nitric oxide (^.^NO) scavenging assay

Nitric Oxide (NO) scavenging was generated from spontaneous decompositions of sodium nitroprusside in 20 mM phosphate buffer (pH 7.4). After incubation of the NO production system, Griess reagent was added and incubated for another 15 min. The absorbance at 540 nm was determined by spectrophotometer [19].

### Hydroxyl radical (^.^OH) scavenging assay

Hydroxyl radicals were generated by a Fenton system (FeSO_2_-H_2_O_2_). When exposed to hydroxyl radicals, the sugar deoxyribose (DR) is degraded to malonaldehyde, which generates a pink chromogen on heating with thiobarbituric acid (TBA) at low pH. The method for determining the scavenging of hydroxyl radicals was performed according to a previously described procedure [20].

### Superoxide and hydrogen peroxide-scavenging activities (SOD/CAT-like activities)

Superoxide radical scavenging based on generation of superoxide radical (O^−^_2_) in the presence of NBT (0.24 mM), glycine buffer (50 mM), EDTA (0.1 mM), and (pH 10.2) is compared with the effect of the compounds tested [21].

Catalase-like activity was determined by the concentration of H_2_O_2_ (0.02 mM) in phosphate buffer (pH 7.0) with absorbance at 240 nm, following the method based on Aebi [22].

### *In vivo* experiments

Animals. Male Swiss mice (25–30 g) were kept in a controlled-temperature room (mean temperature – standard deviation, 21°C – 2°C) with light/dark cycles of 12 hours each and were allowed free access to food (Purina chow) and water. Experimental protocols and procedures were approved by the Federal University of Sergipe Animal Care and Use Committee (no. 56/09).

### Acetic acid writhing reflex test

This study was performed according to Koster et al. [23]. Mice (n = 8, per group) were pretreated with RMO (50, 100 or 200 mg.kg^−1^, p.o.), acetylsalicylic acid (Aspirin, 200 mg.kg^−1^), and the vehicle (saline + Tween-80 0.2%) by oral route (p.o.). Then, after1 h, the mice received the 0.85% acetic acid injection (i.p.). The writhing was counted for 15 min after a latency period of 5 min.

### Formalin-induced nociception test

The procedure described by Hunskaar and Hole [24] was used. Nociception was induced by injecting 20 μl of 1% formalin in distilled water in the subplantar of the righthind paw. Mice (n = 8, per group) previously received RMO (50, 100 or 200 mg.kg^−1^, p.o.), aspirin (200 mg.kg^−1^), and vehicle 1 h prior to injecting formalin. These mice were individually placed in a transparent plexiglass cage observation chamber (25 cm × 15 cm × 15 cm). The amount of time spent licking the injected paw was indicative of pain. The number of lickings from 0 to 5 min (early phase) and 15 to 30 min (late phase) were counted after injection of formalin [24].

### Evaluation of the motor activity

Initially, the mice able to remain on the Rota-rod apparatus (AVS®, Brazil) longer than 180 s (9 rpm) were selected 24 h before the test [25]. Then, the selected animals were divided into five groups (n = 8, per group) and treated p.o. with vehicle (control) or RMO (50, 100 or 200 mg.kg^−1^). Each animal was tested on the rota-rod apparatus and the time (s) they remained on the bar for up to 180 s was recorded after 60, 120 and180 min.

### Carrageenan-induced edema test

Acute hind paw edema was produced by injecting 0.05 mL of carrageenan (1%, prepared as a suspension indistilled water plus Tween 80 at 0.2%) locally into the sub plantar aponeurosis of the right hind paw of mice [26]. Animals were divided into five groups, six rats per group. RMO was administered p.o. at different doses (50, 100, and 200 mg.kg^−1^), indomethacin, standard drug, (INDO, 10 mg.kg^−1^, p.o.), and vehicle (p.o.) was given to a control group. RMO and INDO were administered 1 h prior to injection of carrageenan [27]. Paw volume was measured by dislocation of the water column of a plethysmograph (model EFF 304, Insight®, Brazil) immediately after carrageenan application (time zero) and at 1, 2, 3, 4, 5 and 6 h after its administration.

### Statistical analyses

The parameters data were evaluated by one-way ANOVA (analysis of variance) followed by the Tukey test. Differences were considered to be statistically significant when *p < 0*.05. Data were evaluated using GraphPadPrism version 5.0 (GraphPad Prism Software Inc., San Diego, CA, USA).

## Results and discussion

**Figure 1:**
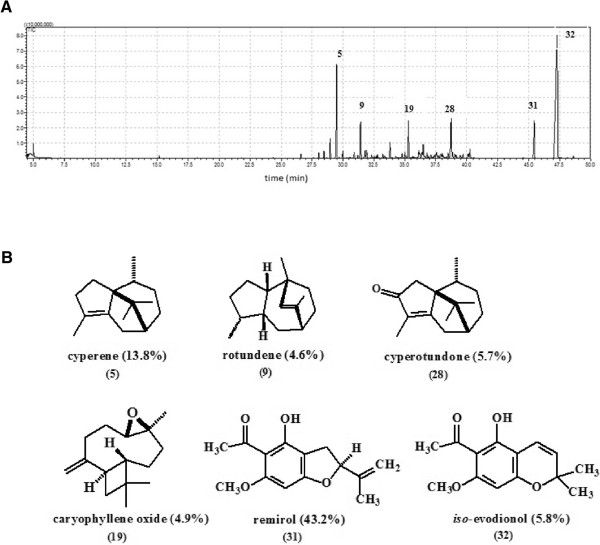
**A. GC/MS chromatogram of essential oil from roots and rhizomes of** ***R. maritima*** **.** Peak numbers corresponding to Table 1. **B.** Chemical structures of major compounds identified in the essential oil of *R. maritima*. Peak numbers corresponding to Table 1.

Hydrodistillation of the rhizomes and roots of *R. maritima* gave a yellow-orange essential oil (RMO), with a yield of 0.08% (w/w). As shown in Table 1, it was possible to identify 32 compounds. The major compounds identified was remirol (43.2%) as previously determined by Siani et al. [11]. However, in this work was possible verify that other compounds with concentration superior to 4% such as cyperene (13.8%), *iso*-evodionol (5.8%), cyperotundone (5.7%), caryophyllene oxide (4.9%), and rotundene (4.6%) were present in the essential oil (Figure 1A and B). Fractions 1–3 were identified by comparing their NMR (^1^H and ^13^C) (see Additional file 1) and GC/MS data with those reported in the literature [11, 28] as *iso*-evodionol, remirol and cyperotundone, respectively. This is the first report on isolation of cyperotundone from *R. maritima.*

**Table 1:** **Essential oil composition of** ***R. maritima***

	Compound	^a^ RI _calc._	^b^ RI	% Peak area
1	1,8-cineole	1030	1026	0.2
2	silphiperfol-4,7(14)-diene	1363	1358	0.8
3	α-copaene	1375	1374	0.8
4	β-elemene	1390	1389	2.3
5	cyperene	1405	1398	13.8
6	(*E*)-caryophyllene	1419	1417	0.8
7	aromadendrene	1447	1439	0.6
8	α-humulene	1456	1452	0.3
9	rotundene	1462	1457	4.6
10	γ-gurjunene	1474	1475	0.9
11	*trans-* cadina-1(6),4-diene	1477	1476	0.8
12	β-selinene	1489	1489	0.3
13	germacrene A	1504	1504	0.3
14	δ-cadinene	1517	1522	0.3
15	α-bulnesene	1504	1509	0.3
16	α-agarofuran	1536	1537	1.9
17	guaia-6,9-dien-4β-ol	1567	1565	0.4
18	acora-3,5-dien-11-ol	1574	1574	0.5
19	caryophyllene oxide	1583	1582	4.9
20	humulene epoxide II	1610	1608	0.9
21	acora-2,4(15)-dien-11-ol	1613	1616	0.5
22	caryophylla-2(12),6-dien-5β-ol	1633	1633	0.5
23	cedr-8(15)-en-10-ol	1651	1652	0.4
24	selin-11-en-4α-ol	1659	1658	0.5
25	14-hydroxy-9-*epi*-(*E*)-caryophyllene	1970	1668	0.5
26	mustakone	1674	1676	0.4
27	germacra-4(15),5,10(14)-trien-1α-ol	1690	1685	0.6
28	cyperotundone	1698	1695^d^	5.7
29	14-hydroxy-humulene	1719	1713	0.7
30	6-isopropenyl-4,8a-dimethyl-3,5,6,7,8,8a-hexahydro-2(1H)-naphtalenone	1789	1790	0.5
31	*iso*-evodionol	1991	1962^c,d^	5.8
32	remirol	2047	2046^c,d^	43.2
	**Monoterpenes**			**0.2**
	**Sesquiterpenes**			**45.8**
	**Benzenoids**			**49.0**
	**Total Identified Compounds**			**95.0**

^a^RI_calc._, retention indices on DB-5MS column calculated according to ref. [14].^b^RI, retention indices according to ref. [15].^c^retention index according to ref. [11]. ^d^co-injection with authentic standard.

**Figure 2:**
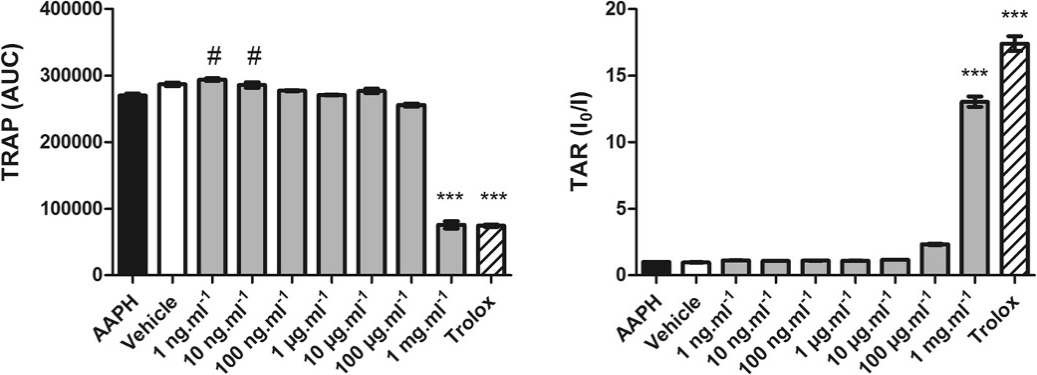
**Total reactive antioxidant potential (TRAP) and total antioxidant reactivity (TAR).** **(A)** TRAP analysis. A free radical source (AAPH) generates peroxyl radical at a constant rate, and the effect of different concentrations of RMO on free radical induced chemiluminescence is measured as area under curve during 60 min. **(B)** TAR values are calculated as the ratio of light intensity in absence of samples (A_O_)/light intensity right after RMO addition **(A)** and expressed as percent of inhibition. All groups denote samples in the presence of AAPH. Bars represent mean ± SEM. ^***^p < 0.0001 antioxidant and # pro-oxidant effect (1-way ANOVA followed by Tukey’s multiple comparison post test).

The TRAP and TAR methods are widely employed to estimate the general antioxidant capacity of samples *in vitro*[29]. These methods were determined using a method based on the quenching of luminol enhanced chemiluminescence derived from the thermolysis of a water-soluble azo compound, AAPH, used as a reliable and quantify able source of alkyl peroxyl radicals. At the TRAP assay, RMO concentration of 1 mg.mL^−1^ showed significant anti-oxidant effects and at concentration of 1 and 10 ng.mL^−1^ RMO showed pro-oxidant effect (Figure 2A). RMO at 1 mg.mL^−1^ also showed significant anti-oxidant capacity in TAR measurement (Figure 2B).

**Figure 3:**
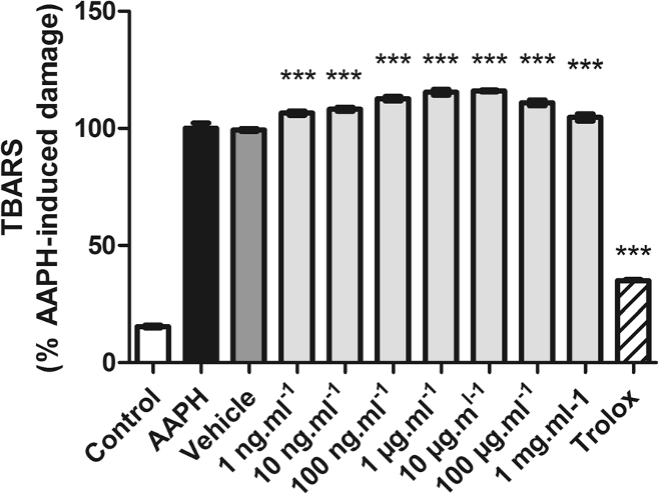
**Thiobarbituric acid-reactive substances (TBARS)** ***in vitro*** **.** A lipid-rich system was incubated with a free radical source (AAPH) and the effect of different concentrations of RMO on the lipoperoxidation was measured by quantifying TBARS. Control is incubation medium without AAPH; other groups contained AAPH alone or in the presence of different concentrations of RMO or its vehicle alone. Bars represent mean ± SEM. *p < 0.05, **p < 0.0001 (1-way ANOVA followed by Tukey’s multiple comparison post test).

**Figure 4:**
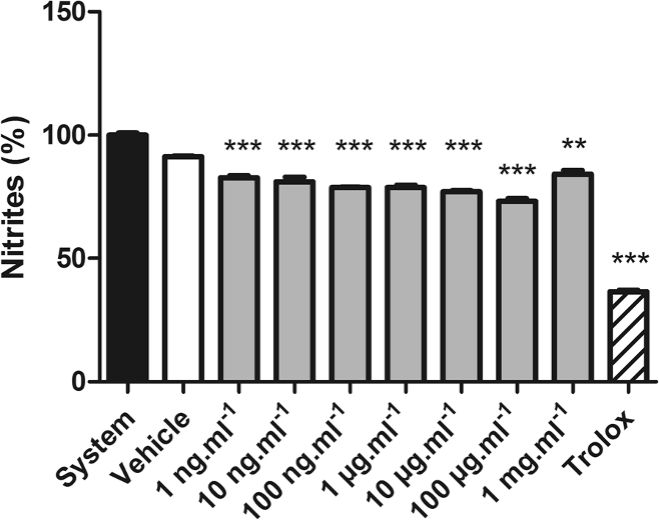
**Nitric oxide radical scavenging activity of RMO.** Values represent mean ± standard error; experiments were performed in triplicate. Different concentrations of the RMO were incubated along with sodium nitroprusside. ***p < 0.001 versus sodium nitroprusside (analysis of variance followed by Tukey test).

Lipid peroxidation has been defined as the biological damage caused by free radicals that are formed under oxidative stress [30]. The ability of RMO to prevent lipid peroxidation was measured by quantifying thiobarbituric acid-reactive substances (TBARS) generated by AAPH in a lipid-rich incubation medium. The effect of different concentrations on lipid peroxidation is shown in Figure 3. Concentrations of RMO from 1 ng.mL^−1^ to 100 μg.mL^−1^ enhanced the AAPH-induced lipoperoxidation.In this study, the ROM was checked for its inhibitory effect on nitric oxide production by the method of Griess. NO radical generated from sodium nitroprusside at physiological pH was found to be inhibited by ROM, that showed scavenging effect upon SNP-induced NO production at all concentrations (Figure 4).

**Figure 5:**
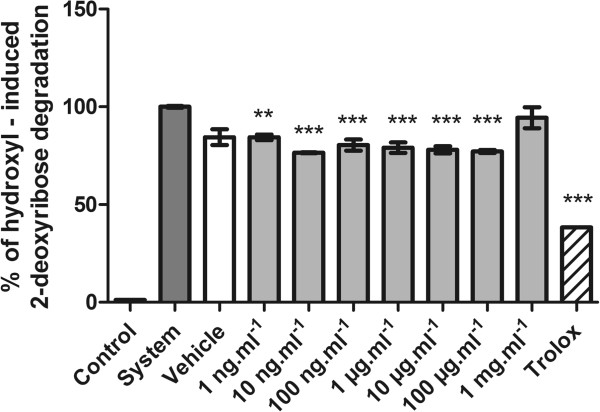
**Hydroxyl radical–scavenging activity of RMO.** Values represent mean ± standard error; experiments were performed in triplicate. **p < 0.01; ***p < 0.001 versus system (analysis of variance followed by Tukey test).

The hydroxyl radical–scavenging capacity of a substance is directly related to its antioxidant activity. This radical has the capacity to join nucleotides in DNA and cause strand breakage, which contributes to carcinogenesis, mutagenesis, and cytotoxicity [31]. We observed that the RMO reduced deoxyribose oxidative damage, induced by the Fenton reaction induction system, at concentrations from 1 ng.mL^−1^ to 100 μg.mL^−1^ (Figure 5).

**Figure 6:**
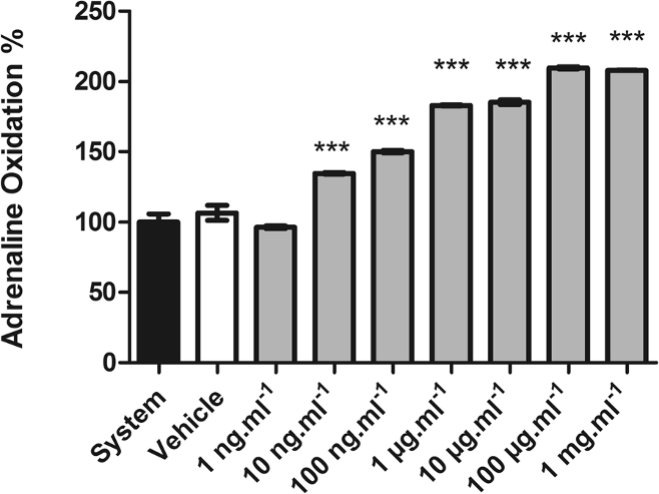
**SOD-like activity was determined by following formation of adrenochrome in a SOD reaction buffer containing native purified catalase and adrenaline (O**_**2**_^**_**^ **generator group).** Bars represent mean ± SEM, ***p < 0.0001 in relation to system. One-way ANOVA followed by Tukey’s multiple comparison post test was applied to all data.

**Figure 7:**
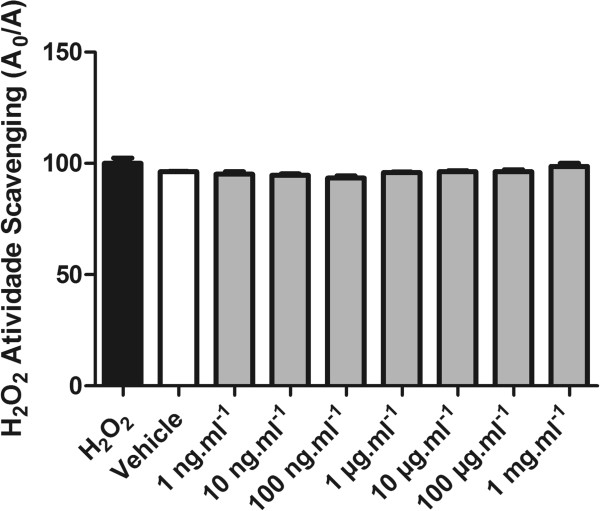
**CAT-like activity was measured in a catalase reaction buffer with H**_**2**_ **O**_**2**_ **.** Bars represent mean ± SEM.

The capacity of RMO to interact with and/or scavenge/quench H_2_O_2_ and superoxide radicals and in vitro was evaluated, respectively, by the catalase-like and the superoxide dismutase-like reaction assays.We observed that RMO caused a significant increase in rate of adrenaline auto-oxidation (Figure 6). On the other hand RMO did not present any scavenging effect in H_2_O_2_ formation in vitro (Figure 7).

The results of this study revealed that RMO has both peripheral and central analgesic properties. The RMO, all doses, orally (p.o.) administered significantly inhibited (p < 0.05, p < 0.01 and p < 0.001) the acetic acid-induced writhings and two phases of formalin-induced nociception in mice (Table 2). Those effects are probably relationship to the inhibition in the peritoneal fluid levels of PGE_2_ and PGF_2α_[32] and with the release inhibition of substance P, and other inflammatory molecules, such as serotonin, histamine, bradykinin, and prostaglandins [33], respectively.

**Table 2:** **Effect of RMO on writhing induced by acetic acid and formalin-induced nociception tests**

Treatment	Dose (mg/kg)	Writhing test	Formalin test	
		Number of writhings ^a^	0-5 min ^a^	15-30 min ^a^
Vehicle	-	32.8 ± 2.8	56.3 ± 5.2	115.0 ± 8.7
RMO	50	20.6 ± 2.3^b^	43.0 ± 2.92	54.5 ± 4.5^d^
RMO	100	18.1 ± 3.6^c^	39.0 ± 3.0	54.8 ± 4.3^d^
RMO	200	10.1 ± 2.5^d^	31.3 ± 5.69^c^	38.6 ± 3.6^d^
Aspirin	200	3.5 ± 1.7^d^	40.1 ± 4.9	35.4 ± 8.9^d^

*n* = 8, per group.

^a^Values represent mean ± S.E.M.

^b^p < 0.05, ^c^p < 0.01 and ^d^p < 0.001 (one-way ANOVA and Tukey’s test), significantly different from control group.

Previous studies suggested that the CNS depression and the non-specific muscle relaxation effect can reduce the response of motor coordination and might invalidate the behavior tests results [34]. We find that all RMO-treated mice, at these doses, did nothave any performance alteration in the rota-rod apparatus (data no shown).

**Figure 8:**
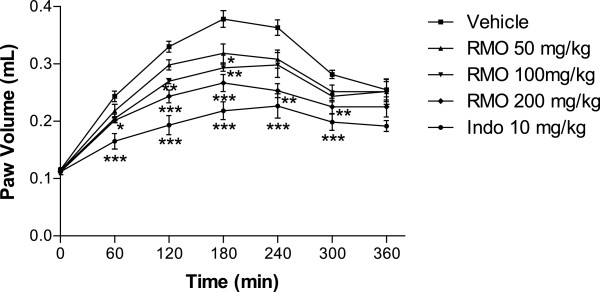
**Effect of RMO or indomethacin (INDO) on mice paw edema induced by carrageenan.** Mice were pretreated with vehicle, INDO (10 mg/kg), or RMO at the doses of 50, 100, and 200 mg/kg (v.o.) 60 min before carrageenan-induced paw edema. Measurements were performed at the times 0, 1, 2, 3, 4, 5 and 6 h after the subplantar injection of carrageenan (1%, 50 μl). Each value represents the mean ± S.E.M. Asterisks denote statistical significance, *P < 0.05, **P < 0.01, and ***P < 0.001, in relation to control group. ANOVA followed by Tukey’s test.

The initial phase of carrageenan paw edema is mediated by histamine and serotonin, while the mediatorsin the later phase are suggested to be arachidonate

metabolites (prostaglandins and leukotrienes) producing an edema dependent on the mobilization of neutrophils [35]. In our experiments, the edematous response was significantly suppressed in mice pretreated with RMO in the first phase of edema, suggesting an inhibitory effect on the release of histamine and/or serotonin (Figure 8). RMO showed a significant inhibition of edema in the second and third phases, suggesting inhibition of 5-lipoxygenase and/or cyclooxygenase, both enzymes involved in the formation of prostaglandins and leukotrienes [34]. This edematous response was also significantly reduced in mice pretreated with indomethacin, a compound known to be a cyclooxygenase inhibitor.

Some studies suggest that antinociceptive and anti-inflammatory effects, such as induced by RMO, could thus also be a consequence of its antioxidant ability, which may prevent the free radical–induced nuclear factor-κB activation and consequent pro-inflammatory cytokines production, a cycle that perpetuates inflammatory processes [30, 36].

## Conclusion

This is the first complete report on the analysis of the volatile constituents from roots and rhizomes and isolation of cyperotundone from *R. maritima*. Their antioxidant activity of the oil essential of the *R. maritima* at 1 mg.mL^−1^ showed significant anti-oxidant capacity in TAR as an increase in AAPH-induced lipoperoxidation at concentrations of from 1 ng.mL^−1^ to 100 μg.mL^−1^, also showing an inhibitory effect on nitric oxide production and in reducing deoxyribose oxidative damage, at concentrations of 1 ng.mL^−1^ to 100 μg.mL^−1^. The antinociceptive and anti-inflammatory activities relevant results demonstrated the essential oil from *R. maritima* observed both peripheral and central analgesic, the acetic acid-induced writhing and two phases of formalin-induced nociception in mice was significant at all doses (p <0.05, p <0.01 and p <0.001), moreover the oil showed a significant inhibition of edema in the second and third stages of the test. The significant antioxidant activities of the essential oil confirm that this species is a natural source of biologically active compounds and could thus also be a consequence of the effects antinociceptive and anti-inflammatory of the oil essential the *R. maritima*. The present study adds more information to the ethnopharmacological knowledge of the plant and corroborates its use in Brazil.

## Electronic supplementary material

Additional file 1: (PDF 1 MB)

## Abbreviations

AAPH: 2,2'-Azobisisobutyramidinium chloride
ANOVA: Analysis of variance
AUC: Area under the curve
CAT: Catalase-like activity
CL: Chemiluminescense
CNS: Central nervous system
DR: Deoxyribose
EDTA: Ethylenediamine tetraacetic acid
GC: Gas chromatography
i. p.: Intraperitoneal
INDO: Indomethacin
MS: Mass spectrometry
NBT: Nitroblue tetrazolium
NO: Nitric oxide
p.o.: per os
RMO: Essential oil from *R. maritima*
ROS: Reactive oxygen species
SNP: Sodium nitroprusside
SOD: Superoxide dismutase
TAR: Total antioxidant reactivity
TBA: Thiobarbituric acid
TBARS: Thiobarbituric acid-reactive substances
TLC: Thin-layer chromatography
TRAP: Total reactive antioxidant potential.
