# Supplementary material for: Chemical composition, antinociceptive, anti-inflammatory and redox properties in vitro of the essential oil from Remirea maritima Aubl. (Cyperaceae)
Source: BMC Complement Altern Med. 2014 Dec 23;14:514. doi: 10.1186/1472-6882-14-514 (PMC4528852; doi:10.1186/1472-6882-14-514)
Supplement: Supplementary file 1 — Additional file 1: (PDF 1 MB) [file 12906_2013_2123_MOESM1_ESM.pdf]

ADDITIONAL FILE 1

Chemical composition, antinociceptive, anti-Inflammatory and redox properties *in vitro* of the essential oil from *Remirea maritima* Aubl. (Cyperaceae)

Alessandra Silva Rabelo[1], Mairim Russo Serafini*[1], Thallita Kelly Rabelo[1], Marcelia Garcez Dória de Melo[1], Douglas da Silva Prado[1], Daniel Pens Gelain[2], José Claudio Fonseca Moreira[2], Marília dos Santos Bezerra[1], Thanany Brasil da Silva[3], Emmanoel Vilaça Costa[3], Paulo Cesar de Lima Nogueira[3], Valéria Regina de Souza Moraes[3], Ana Paula do Nascimento Prata[4], Lucindo José Quintans-Júnior[1], and Adriano Antunes Souza Araújo[1]*

[1] Laboratório de Ensaios Farmacêuticos e Toxicidade, Universidade Federal de Sergipe (LeFT/UFS), 49100-000, São Cristóvão, Sergipe, Brazil

[2]Centro de Estudos em Estresse Oxidativo, Departamento de Bioquímica, Instituto de Ciências Básicas da Saúde, Universidade Federal do Rio Grande do Sul, 13083-970, Porto Alegre, Rio Grande do Sul, Brazil

[3]Laboratório de Pesquisa em Química Orgânica de Sergipe (LABORGANICS), Departamento de Química, Universidade Federal de Sergipe, 49100-000, São Cristóvão, Sergipe, Brazil

[4]Laboratório de Sistemática Vegetal, Departamento de Biologia, Universidade Federal de Sergipe, 49100-000, São Cristóvão, Sergipe, Brazil

---

*e-mail: maiserafini@hotmail.com

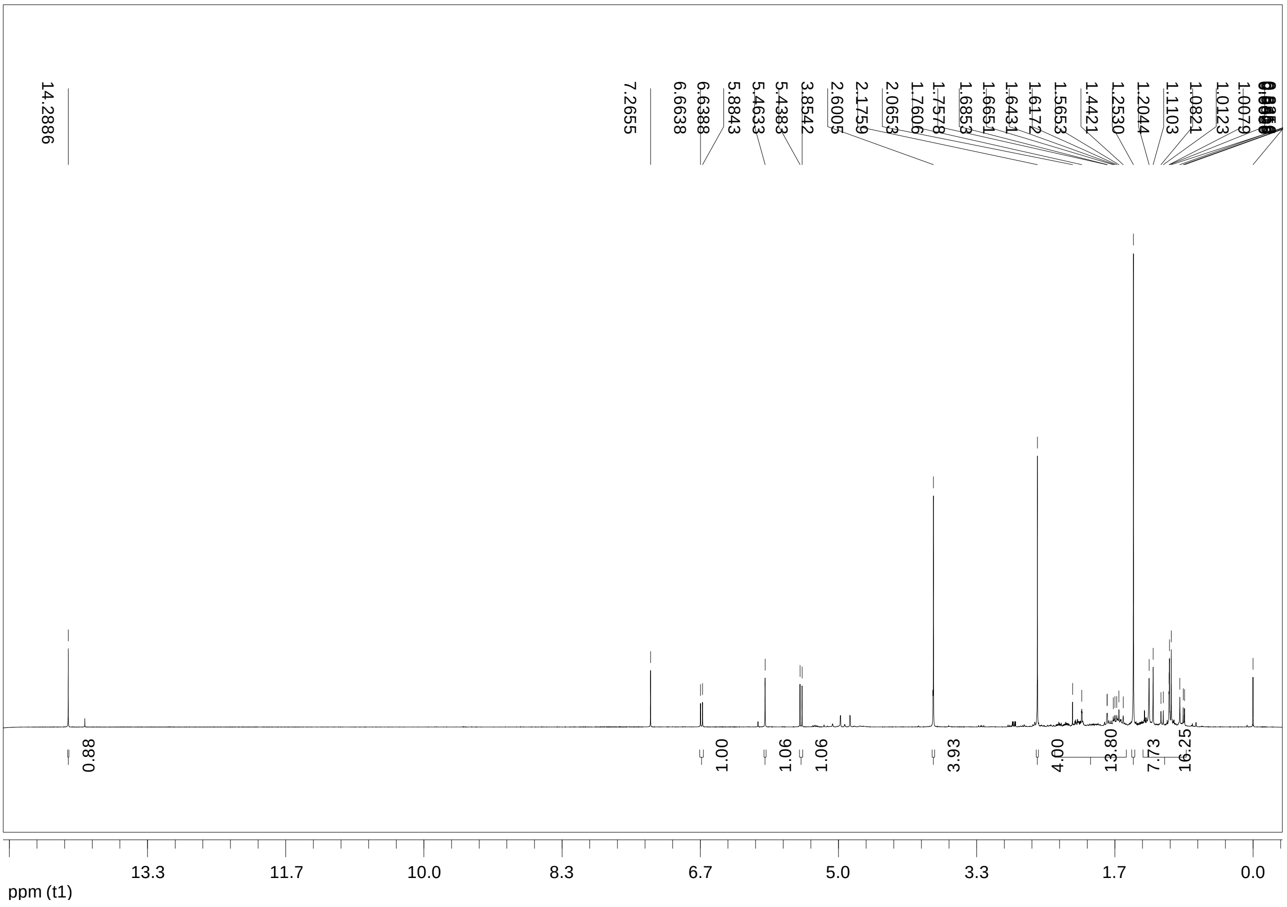

Figure 1S. ^{1}H NMR spectrum of compound **1** (*iso*-evodionol) in $CDCl_3$ at 400 MHz.

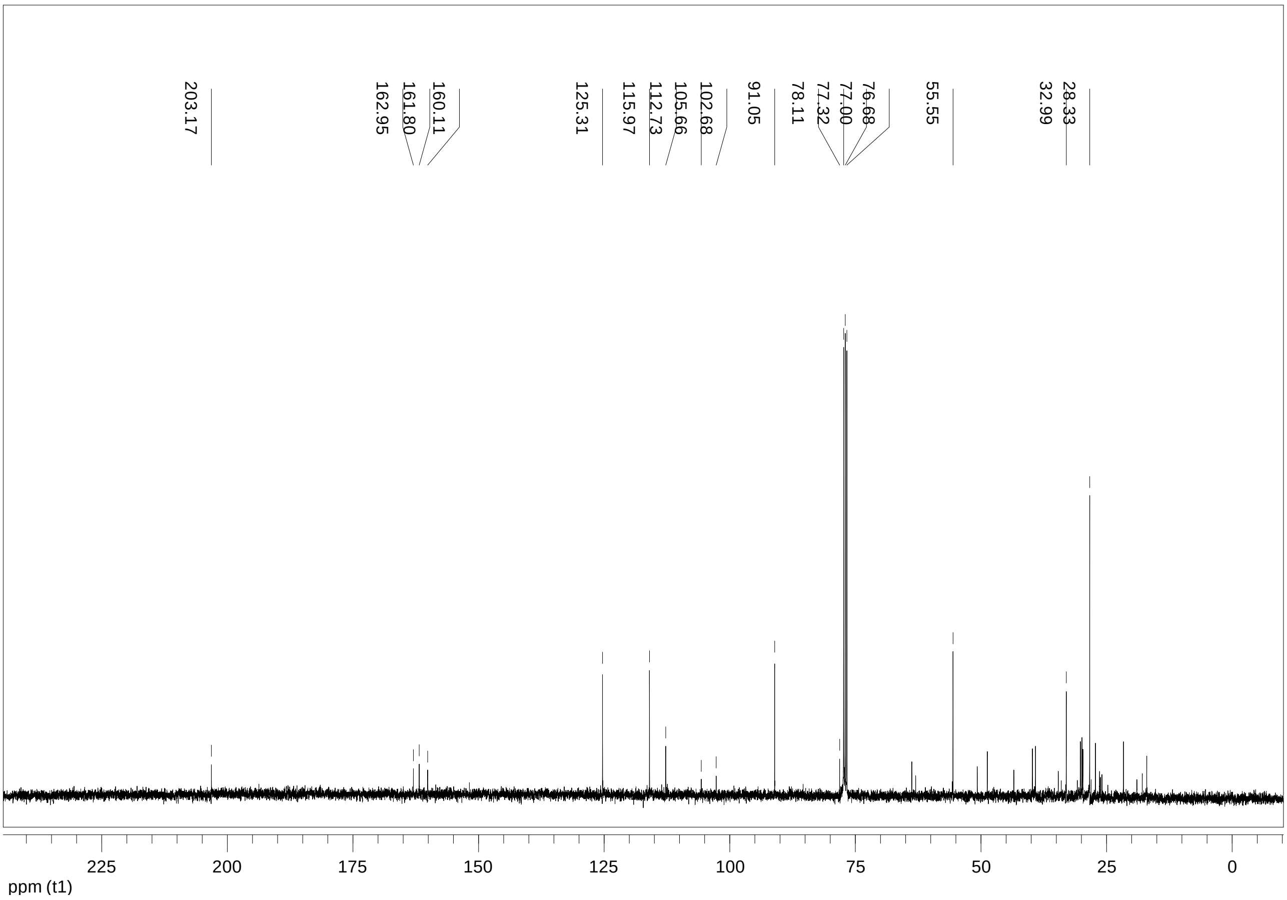

Figure 2S. $^{13}C\{^{1}H\}$ spectrum of compound **1** (*iso*-evodionol) in CDCl at 100 MHz.

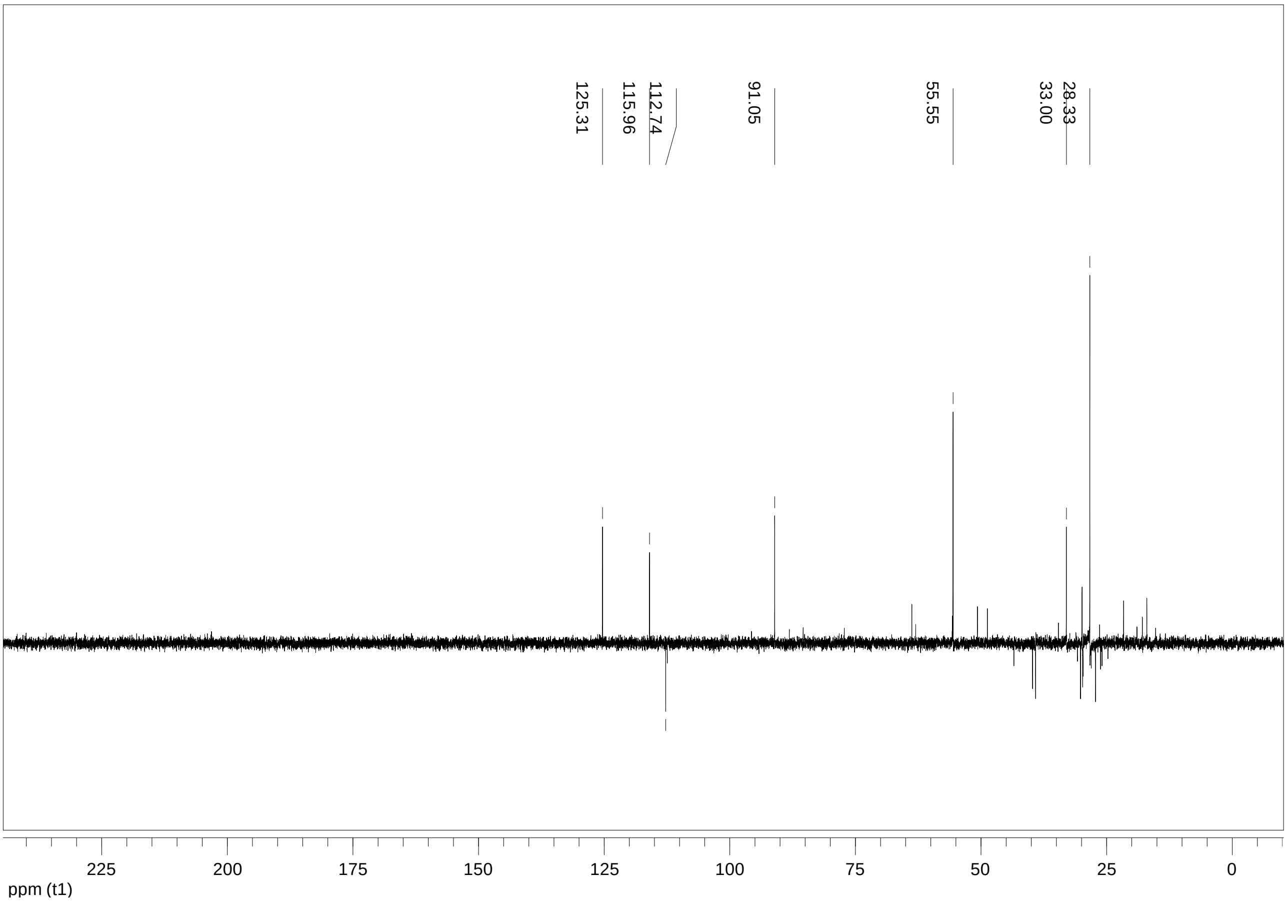

Figure 3S. DEPT 135 NMR spectrum of compound **1** (*iso*-evodionol) in CDCl at 100 MHz.

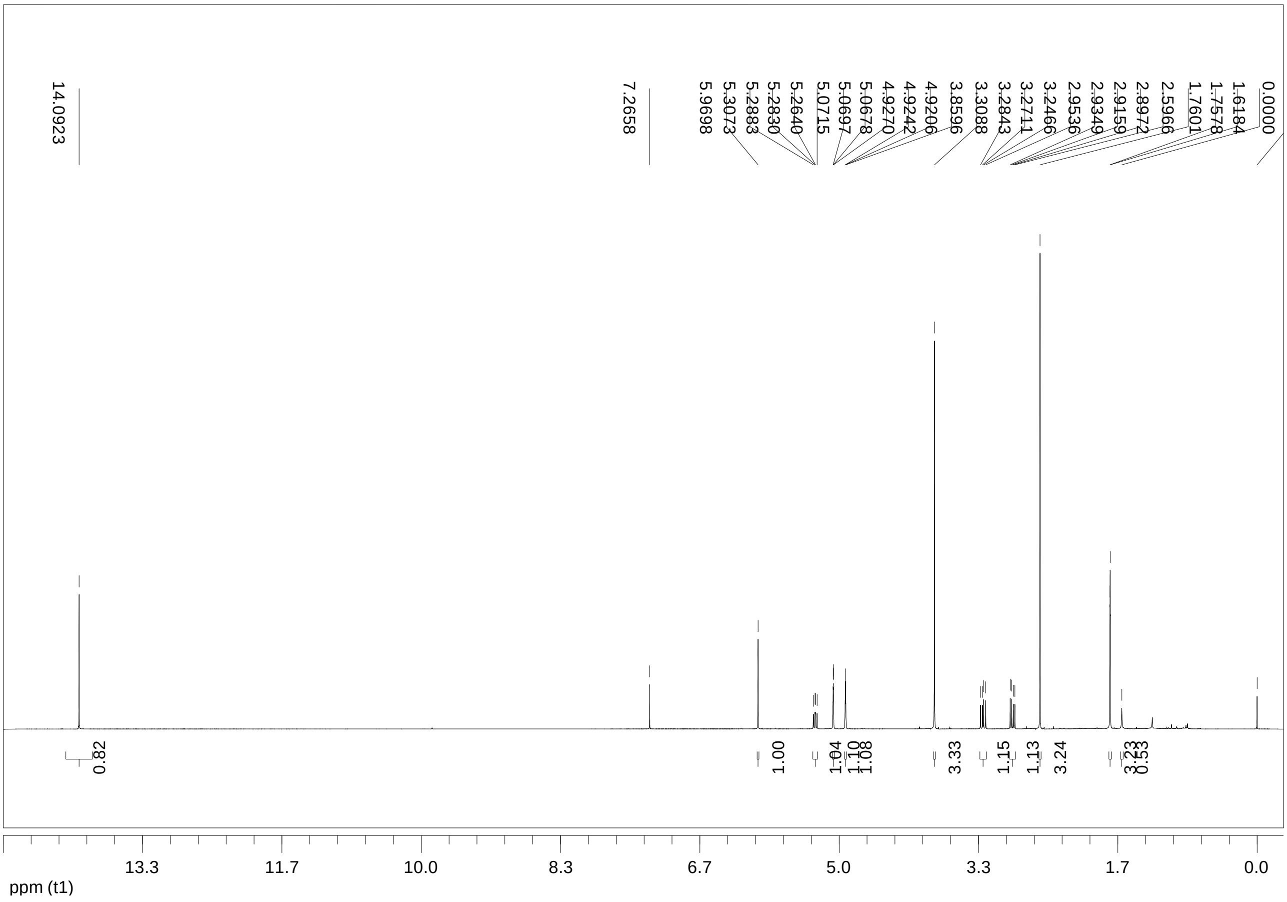

Figure 4S. ^{1}H NMR spectrum of compound **2** (remirol) in $CDCl_3$ at 400 MHz.

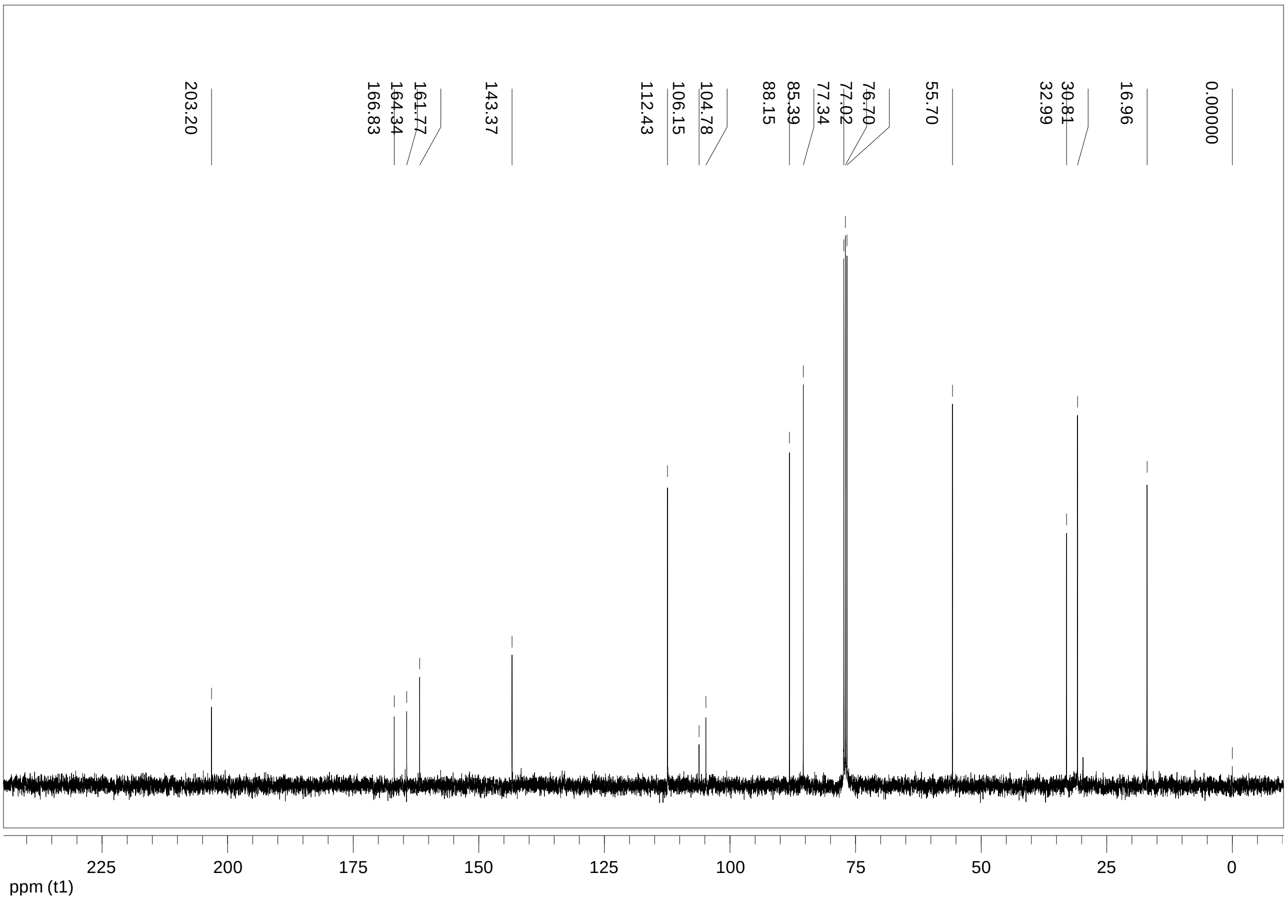

Figure 5S. $^{13}C\{^{1}H\}$ spectrum of compound **2** (remirol) in CDCl at 100 MHz.

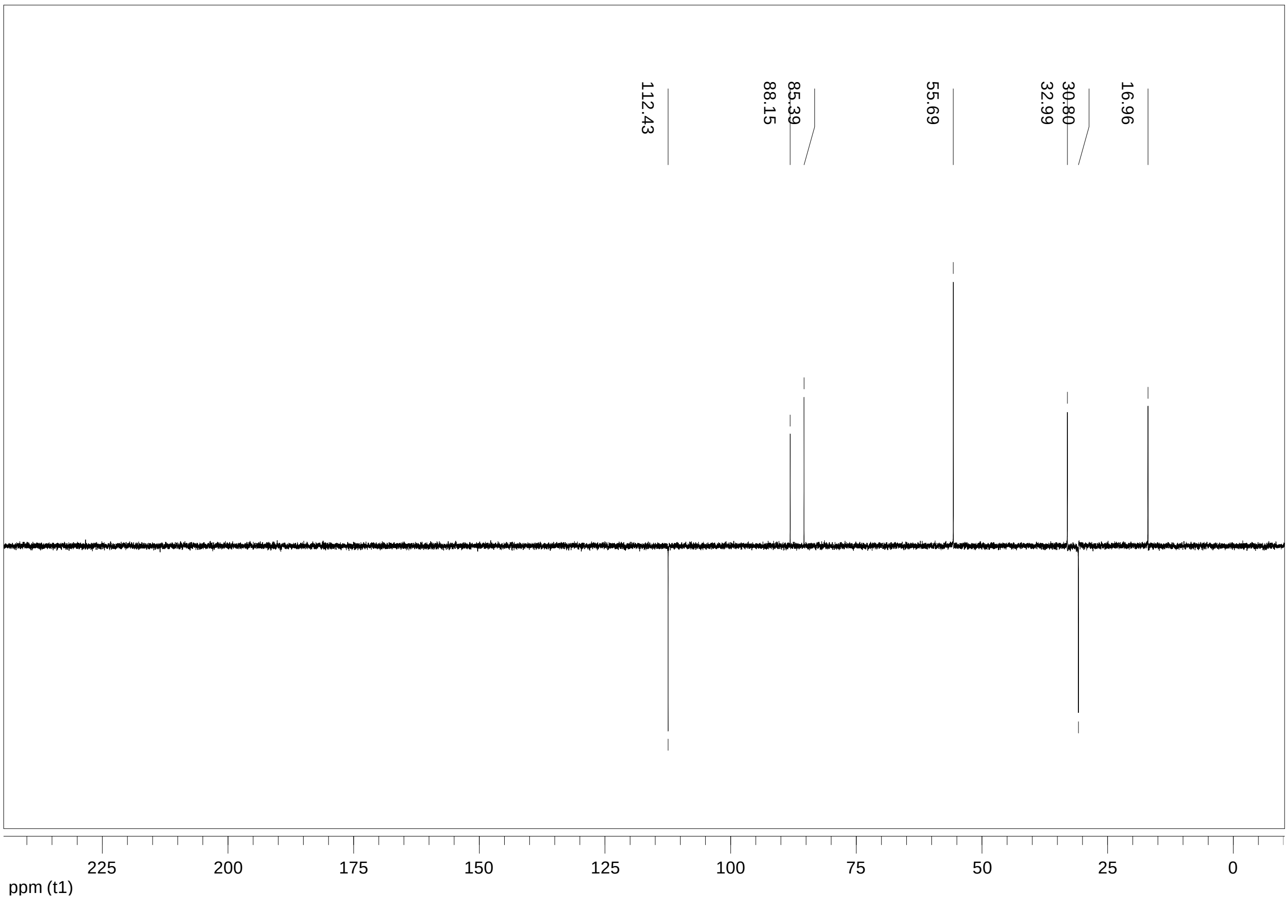

Figure 6S. DEPT 135 NMR spectrum of compound **2** (remirol) in CDCl at 100 MHz.

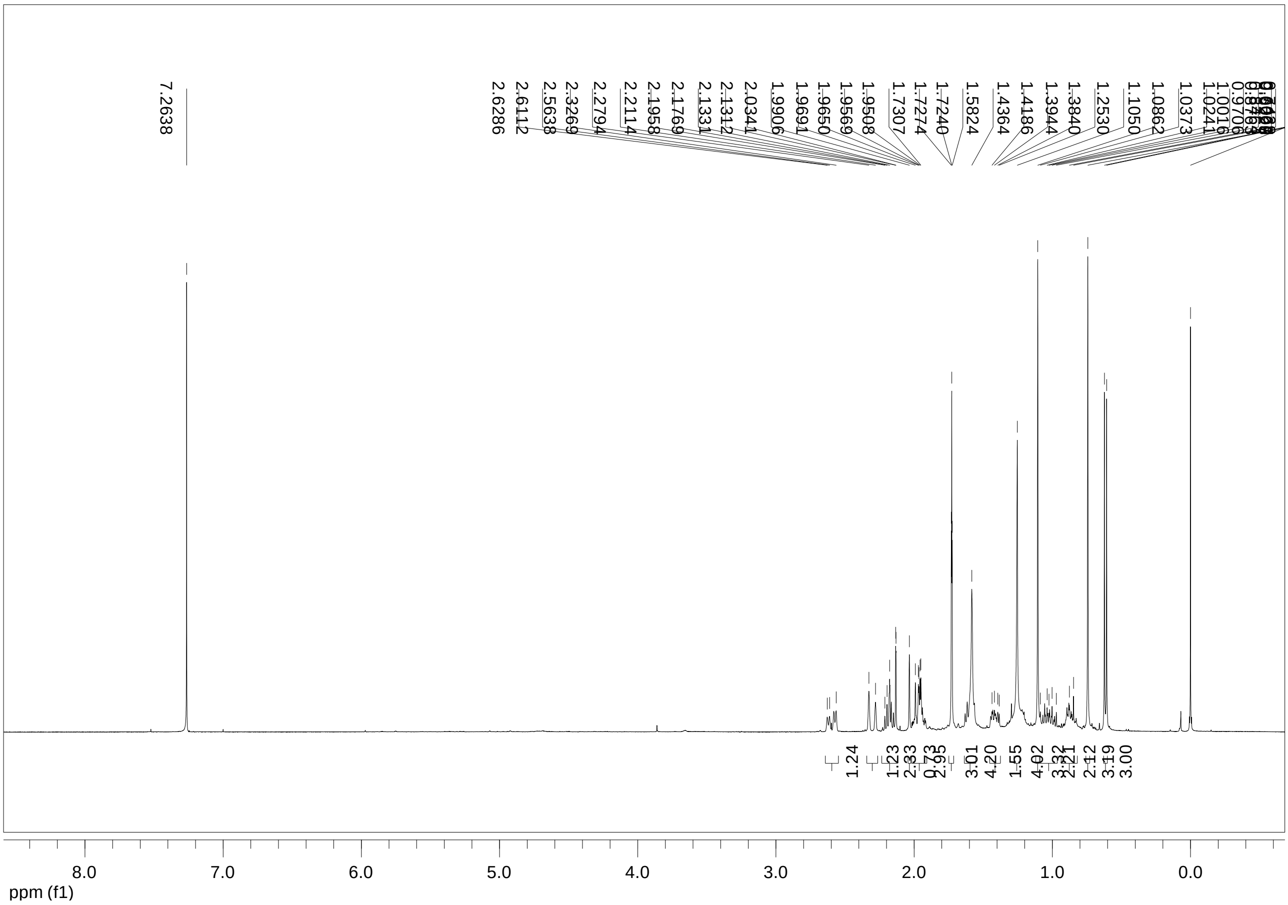

Figure 7S. ^{1}H NMR spectrum of compound **3** (cyperotundone) in $CDCl_3$ at 400 MHz.

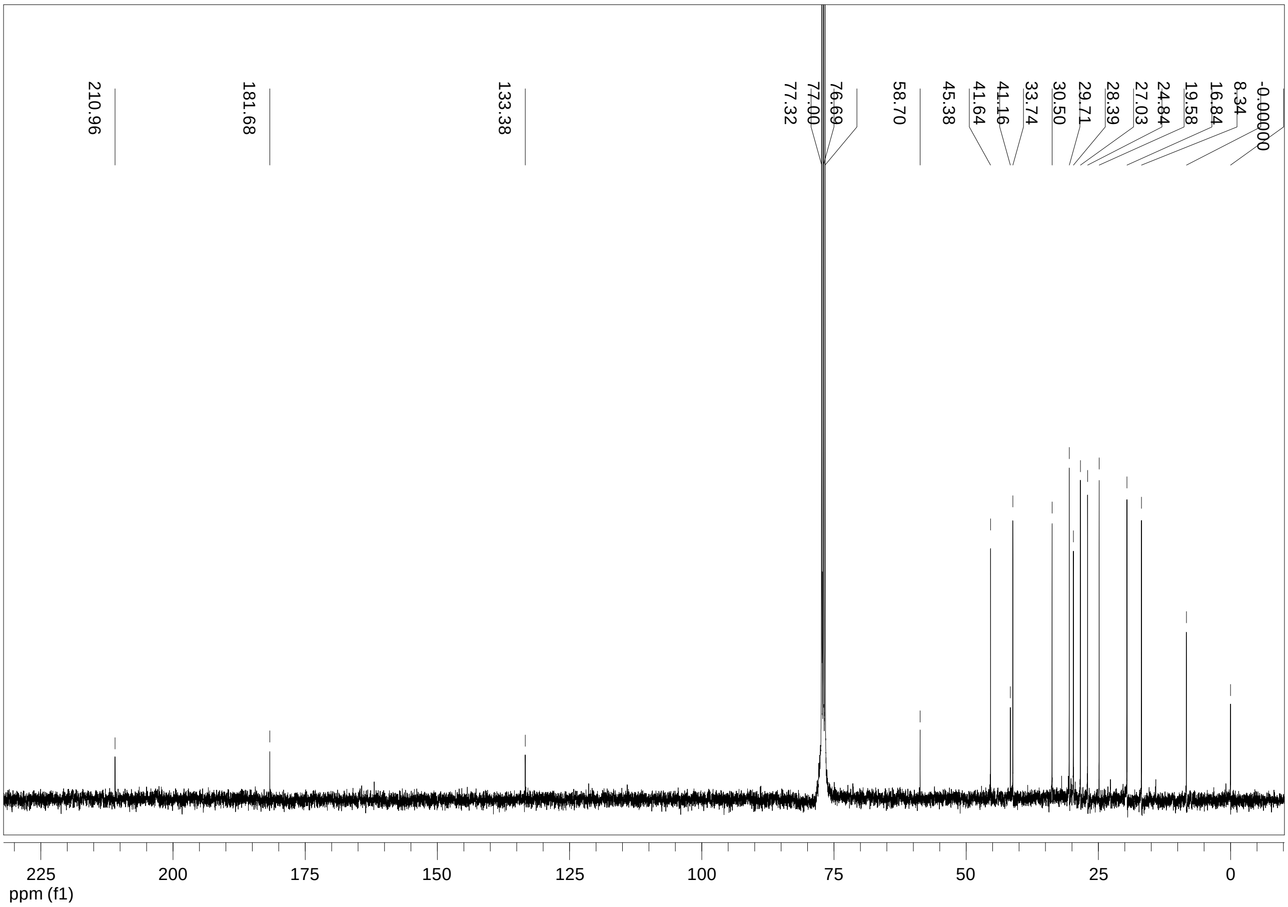

Figure 8S. $^{13}C\{^{1}H\}$ spectrum of compound **3** (cyperotundone) in CDCl at 100 MHz.
